# A Mutation in Synaptojanin 2 Causes Progressive Hearing Loss in the ENU-Mutagenised Mouse Strain *Mozart*

**DOI:** 10.1371/journal.pone.0017607

**Published:** 2011-03-15

**Authors:** Shehnaaz S. M. Manji, Louise H. Williams, Kerry A. Miller, Lisa M. Ooms, Melanie Bahlo, Christina A. Mitchell, Hans-Henrik M. Dahl

**Affiliations:** 1 Genetic Hearing Research, Murdoch Childrens Research Institute, Royal Children's Hospital, Parkville, Victoria, Australia; 2 Department of Paediatrics, Melbourne University, Royal Children's Hospital, Parkville, Victoria, Australia; 3 Department of Biochemistry and Molecular Biology, Monash University, Clayton, Victoria, Australia; 4 Bioinformatics, Walter & Eliza Hall Institute, Parkville, Victoria, Australia; University of Texas MD Anderson Cancer Center, United States of America

## Abstract

**Background:**

Hearing impairment is the most common sensory impairment in humans, affecting 1∶1,000 births. We have identified an ENU generated mouse mutant, *Mozart*, with recessively inherited, non-syndromic progressive hearing loss caused by a mutation in the synaptojanin 2 (Synj2), a central regulatory enzyme in the phosphoinositide-signaling cascade.

**Methodology/Principal Findings:**

The hearing loss in *Mozart* is caused by a p.Asn538Lys mutation in the catalytic domain of the inositol polyphosphate 5-phosphatase synaptojanin 2. Within the cochlea, *Synj2* mRNA expression was detected in the inner and outer hair cells but not in the spiral ganglion. *Synj2* ^N538K^ mutant protein showed loss of lipid phosphatase activity, and was unable to degrade phosphoinositide signaling molecules. Mutant *Mozart* mice (*Synj2* ^N538K/N538K^) exhibited progressive hearing loss and showed signs of hair cell degeneration as early as two weeks of age, with fusion of stereocilia followed by complete loss of hair bundles and ultimately loss of hair cells. No changes in vestibular or neurological function, or other clinical or behavioral manifestations were apparent.

**Conclusions/Significance:**

Phosphoinositides are membrane associated signaling molecules that regulate many cellular processes including cell death, proliferation, actin polymerization and ion channel activity. These results reveal Synj2 as a critical regulator of hair cell survival that is essential for hair cell maintenance and hearing function.

## Introduction

Deafness is an etiologically heterogeneous trait where the age of onset, severity and site of lesion can vary. It can be caused by genetic and/or environmental factors. Environmental risk factors include premature birth, bacterial and viral infections, exposure to loud noise and to ototoxic drugs (which include commonly used aminoglycoside antibiotics such as kanamycin, neomycin and gentamycin, as well as the anticancer drugs cisplatin and methotrexate). However, more than 60% of congenital non-syndromic deafness is caused by mutations in one of an estimated 200 or more “deafness” genes [1]. Inherited hearing losses are usually transmitted as monogenic disorders and most often in an autosomal recessive mode [2], the latter accounts for approximately 80% of non-syndromic inherited hearing loss. The identification of genes that cause deafness when mutated has significantly improved our knowledge about the auditory pathway and led to better genetic counseling and management of people affected by hearing loss. Despite this, there are still large gaps in our knowledge of genes involved in hearing loss and the molecular mechanisms of auditory function.

**Figure 1:**
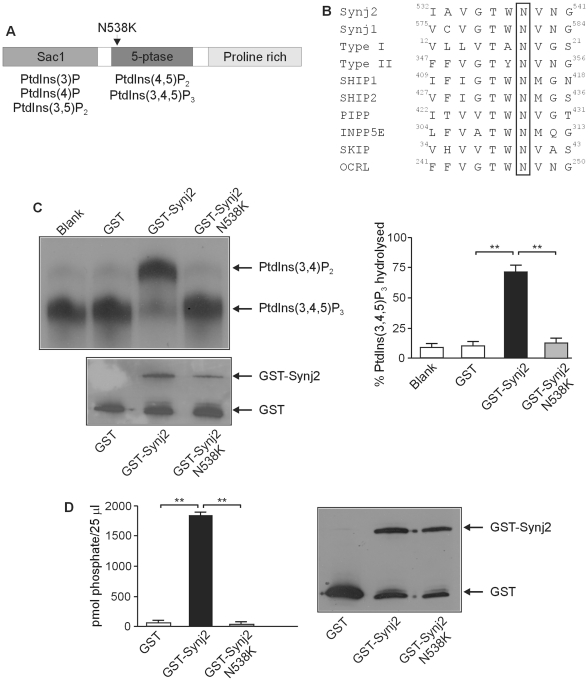
The N538K mutation abolishes Synj2 inositol polyphosphate 5-phosphatase activity. Domain structure of Synj2 indicating the position of the N538K mutation in the 5-phosphatase domain (Panel A). The phosphoinositide substrates hydrolyzed by the Sac1 and 5-phosphatase domains are indicated. Protein sequence alignment of murine 5-phosphatases showing N^538^ (boxed) is conserved in all 10 mammalian 5-phosphatases (Panel B). Accession numbers: Synj2: Q9D2G5, Synj1: NP_001157955, Type I: AAH56341, Type II: CAM16097, SHIP1: Q9ES52, SHIP2: Q6P549, PIPP: AAI31635, INPP5E: NP_149125, SKIP: NP_032942, OCRL: NP_796189. GST-Synj2 wild-type or Synj2^N538K^ mutant 5-phosphatase domains or GST alone were purified from *E. coli* and assayed for PtdIns([^32^P]3,4,5)P_3_ 5-phosphatase activity (Panel C). Lipid products were separated by thin layer chromatography (top left panel). The migration of PtdIns(3,4)P_2_ and PtdIns(3,4,5)P_3_ are indicated. The relative amount of recombinant protein added to each reaction was determined by immunoblotting with GST antibodies (lower left panel). The relative percentage of PtdIns(3,4,5)P_3_ substrate hydrolyzed was determined by densitometry (right panel). Bars represent mean ± SEM from 3 independent experiments. **p<0.001. Recombinant GST-Synj2 wild-type or Synj2^N538K^ mutant 5-phosphatase domains or GST alone were purified from *E. coli* and assayed for PtdIns(4,5)P_2_ 5-phosphatase activity (Panel D). Phosphate release (pmol/25 µl sample) was measured using a malachite green assay (left panel). Bars represent mean ± SEM from 2 independent experiments. The relative amount of recombinant protein added to each reaction was determined by Western blotting with GST antibodies (right panel). **p<0.001.

Phosphoinositides are membrane-bound signaling molecules that control many important cellular processes, such as membrane trafficking, nucleation of actin filaments, dynamin function, and the activity of ion channels and transporters [3], [4], [5]. It has been suggested that phosphoinositides play an important role in regulating cellular processes essential for normal hearing [6], [7], [8], [9], but little is known of how changes in phosphoinositide metabolism may lead to deafness. The levels of phosphoinositide signaling molecules, including phosphatidylinositol 4,5 bisphosphate (PtdIns(4,5)P_2_) and phosphatidylinositol 3,4,5 trisphosphate (PtdIns(3,4,5)P_3_), are controlled by lipid kinases, which synthesize these signaling molecules, and by phosphoinositide phosphatases, which remove phosphate groups from the inositol ring and terminate the signalling function. Synaptojanin 2 (Synj2) belongs to a family of ten inositol polyphosphate 5-phosphatases (5-phosphatases) that remove the 5-position phosphate from phosphoinositides such as PtdIns(3,4,5)P_3_ and PtdIns(4,5)P_2_ forming PtdIns(3,4)P_2_ and PtdIns(4)P, respectively [10], [11], [12]. Synj2 and it's closest family member, Synj1, contain two distinct catalytic domains, a central 5-phosphatase domain and an N-terminal Sac1 domain which degrades PtdIns(3,4)P_2_, PtdIns(4)P and PtdIns(3)P to PtdIns (Fig. 1A) [10]. A C-terminal proline-rich domain interacts with specific combinations of partner proteins, interactions that affect intracellular localization of Synj2 [13]. *Synj1* knockout mice die shortly after birth, exhibiting neurological defects, correlating with increased levels of PtdIns(4,5)P_2_ and an accumulation of clathrin-coated vesicles (CCVs) in nerve terminals [14], [15]. Defects in *Synj1* also cause slowed endocytosis, depletion of synaptic vesicles, accumulation of CCVs and aggregation of cortical actin at neuromuscular junction synapses in *C.elegans*, *Drosophila* and *zebrafish* [16], [17], [18], [19]. Although no mutant *Synj2* mice have so far been reported, it has been proposed *Synj2* may represent a candidate gene for male mouse sterility [20].

To identify novel genes that contribute to deafness we undertook a phenotypic driven approach in which mice at the Australian Phenomics Facility (APF) were ENU mutagenised and progeny were screened for recessively inherited hearing loss. In one of our strains, *Mozart*, we have identified the causative mutation to be in the *Synj2* gene, which has not previously been associated with deafness. The causative mutation in *Synj2* is located in a critical catalytic residue in the 5-phosphatase domain and results in loss of 5-phosphatase activity. In *Mozart* this mutation leads to gradual hearing loss accompanied by hair cell degeneration. This study identifies *Synj2* as a critical regulator of hair cell survival that is essential for maintaining normal hearing.

## Results

### A *Synj2* Mutation is Responsible for Hearing Loss in the *Mozart* Mouse

The *Mozart* mouse was identified in a mouse ENU mutagenesis program in which G3 offspring of ENU-mutagenised mice were screened for recessively inherited hearing loss. To identify the causative mutation in the *Mozart* mouse, genome-wide microsatellite marker analysis on DNA from 20 affected F2 mice was completed. The results showed linkage to D17Mit113 on chromosome 17 with a LOD score of 4.5. Fine mapping of 69 affected mice using amplifluor SNP assays narrowed the candidate region to a 2.1Mb interval between rs13482843 and rs13482851. The coding regions of all 8 known protein coding genes within this region were sequenced and the only change found was in the *Synj2* gene. The causative mutation in *Mozart* is a T to A nucleotide change at nucleotide 1641 (c.1641T>A) in the *Synj2* mRNA (Ensembl transcript ENSMUST00000061091). This results in a p.Asn538Lys (N538K) substitution (UniProtKB/Swiss-Prot database Q9D2G5) in the highly conserved catalytic 5-phosphatase domain of the enzyme (Fig. 1A and 1B). Previous studies have predicted that this Asn orientates a catalytic Asp by forming a hydrogen bond and mutation of this residue results in a complete loss in the ability of INPP5A to hydrolyze Ins(1,4,5)P_3_ [21], [22]. To investigate the effect of the N538K mutation on Synj2 phosphatase activity, GST-tagged wild-type and mutant Synj2^N538K^ 5-phosphatase domains were expressed in *E. coli*, purified and analyzed for 5-phosphatase catalytic activity in hydrolyzing PtdIns(3,4,5)P_3_ to form PtdIns(3,4)P_4_. While the wild-type Synj2 effectively hydrolysed PtdIns(3,4,5)P_3_ generating PtdIns(3,4)P_2_, the Synj2^N538K^ construct exhibited no 5-phosphatase activity (Fig. 1C). Similarly, wild-type Synj2 degraded PtdIns(4,5)P_2_ forming PtdIns(4)P, but the Synj2^N538K^ mutant construct exhibited no 5-phosphatase activity at the same protein concentration (Fig. 1D). Therefore in Synj2, like other 5-phosphatase family members, mutation of the conserved Asp^538^ residue results in loss of 5-phosphatase catalytic activity.

### *Synj2* ^N538K/N538K^ Mice Show a Progressive Sensorineural Hearing Loss without Vestibular Dysfunction

**Figure 2:**
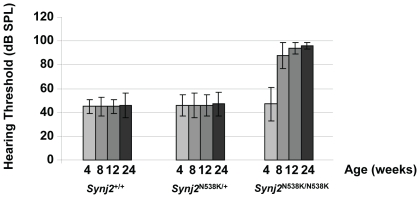
*Synj2* ^N538K/N538K^ mutant mice show progressive age-related hearing loss. Hearing thresholds (dB SPL) in *Synj2* ^+/+^, *Synj2* ^N538K/+^ and *Synj2* ^N538K/N538K^ mice at age 4, 8, 12 and 24 weeks. Hearing threshold values shown are averages of at least 20 mice per genotype and error bars represent the standard deviation.

Auditory function was analyzed in *Synj2* ^N538K/N538K^ mice by measuring auditory brainstem response (ABR). *Synj2* ^N538K/N538K^ mice are born with normal hearing, but their hearing deteriorated by 8 weeks. By 12 weeks the mice were severely deaf. Fig. 2 shows the hearing profiles of *Synj2* ^+/+^, *Synj2* ^N538K/+^ and *Synj2* ^N538K/N538K^ mice at 4, 8, 12 and 24 weeks. A minimum of 20 mice were included in each group. Heterozygous mice showed a similar audiological profile as wild-type mice. No circling or head tossing/tilting behavior indicative of a vestibular dysfunction was observed in *Synj2* ^N538K/N538K^ mice. When we examined the hearing in F2 progeny from the C57BL/6 *Synj2* ^N538K/N538K^ outcross with the CBA/H mapping strain we noted that a milder deafness phenotype was evident in 25% of the offspring. The hearing thresholds in these mice would be approximately 30dB lower than the C57BL/6 *Synj2* ^N538K/N538K^ mice. This was not the case if *Synj2* ^N538K/N538K^ mice were intercrossed with a BALB/c strain, suggesting the interaction of one or more modifier genes from the C57BL/6 background. Genotyping showed that the cadherin 23 Ahl variant (Cdh23^ahl^) is responsible for modifying the severity of the *Synj2*-associated hearing loss (results not shown).

### Neurological and Behavioral Studies in *Synj2* ^N538K/N538K^ Mice

**Figure 3:**
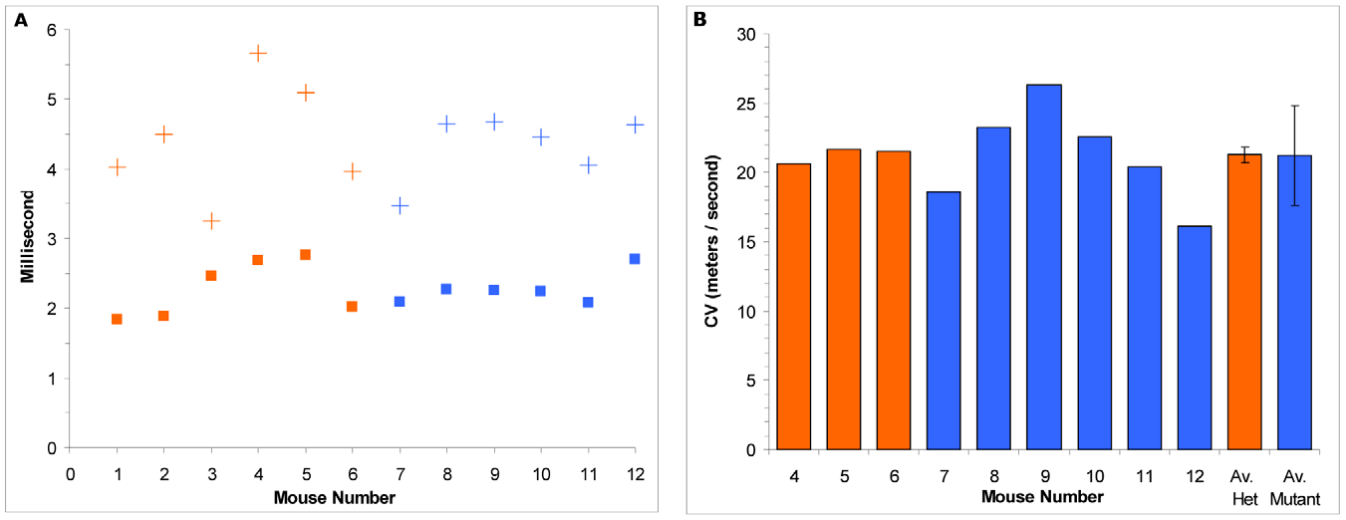
*Synj2* ^N538K/N538K^ mice exhibit normal nerve conduction. Measurements of the time from application of a sciatic stimulus pulse to the first (squares) and second (crosses) muscle response (Panel A). Mice numbered 1–6 (orange) are *Synj2* ^+//N538K^ littermates and mice number 7–12 (blue) are *Synj2* ^N538K/N538K^ mutants. Conduction velocity (CV) of motor nerves to the gastrocnemius muscle (Panel B). The Average CV is shown (Av) and error bars represent the standard deviation in the 3 *Synj2* ^+/N538K^ and 6 *Synj2* ^N538K/N538K^ mice.

As mutations in genes regulating phosphoinositide signalling pathways are often associated with neurological features [12], [23], we examined peripheral nerve conductance in *Synj2* ^N538K/N538K^ mice. Fig. 3A shows the time from application of the sciatic stimulus pulse to the first and second muscle response in 6 *Synj2* ^N538K/+^ and 6 *Synj2* ^N538K/N538K^ mice. Much of the variation in these data is likely to be due to a slight variation in the position of the stimulating electrode along the sciatic nerve. It is clear however, that there was no systematic variation in the latency between *Synj2* ^N538K/+^ and *Synj2* ^N538K/N538K^ mice. Nerve conductance was also examined directly by determining the difference in latency of the first peak when changing the point of nerve stimulation by a known distance. This was analyzed in mice used previously in the nerve conductance test (numbers 4 to 12). The conduction velocities range from 16–23 meters/second across the 8 mice and no significant differences between *Synj2* ^N538K/+^ and *Synj2* ^N538K/N538K^ mice were detected (Fig. 3B).

Primary behavioral screens of *Synj2* ^+/+^ and *Synj2* ^N538K/N538K^ mice consisting of locomotor, light dark test, Y-maze, tail suspension, hot plate and marble burying tests, did not reveal any significant differences between wild-type and *Synj2* ^N538K/N538K^ mice.

The mutant *Mozart* mice have a normal life span, are fertile and, apart from the hearing loss, do not appear to have any additional clinical or behavioural manifestations.

### Degeneration of Hair Cell Structure and Stereocilia Morphology in *Synj2* ^N538K/N538K^ Mice

**Figure 4:**
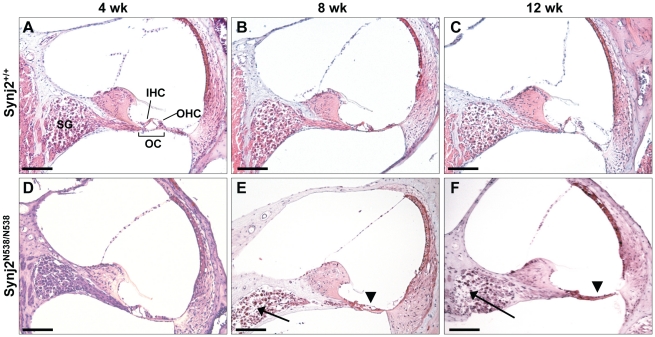
Synj2^N538K/N538K^ cochleae exhibit degeneration. Synj2^+/+^ (Panels A, B and C) and Synj2^N538K/N538K^ (Panels D, E and F) cochleae were sectioned at 4 (Panels A and D), 8 (Panels B and E) and 12 (Panels C and F) weeks of age and H&E stained. Representative cochleae are shown. Synj2^N538K/N538K^ cochleae show signs of degeneration at 8 and 12 weeks of age, with evidence of collapse of the organ of Corti (arrow head, panels E and F) and degeneration of spiral ganglion neurons (arrow, panels E and F). SG, spiral ganglion; OC, organ of corti; OHC, outer hair cells; IHC, inner hair cell. Scale bar: 100 µm.

Examination of outer and middle ear structures did not identify any visible structural changes in *Synj2* ^N538K/N538K^ mice compared to wild-type mice. Gross structural changes in the inner ear were initially examined by hematoxylin and eosin (H&E) staining of cochlear sections. We observed a gradual loss of inner and outer hair cells in 8 and 12 week old mutant mice, starting in the basal turn of the cochlea and spreading towards the apical region, leading to a collapse of the Organ of Corti (Fig. 4). At the same time there was a reduction in the number of spiral ganglia cells and the remaining cells showed increased vacuolation. *Synj2* ^N538K/+^ mice resembled wild-type mice, showing no signs of hair cell degeneration or loss of spiral ganglion neurons (data not shown).

**Figure 5:**
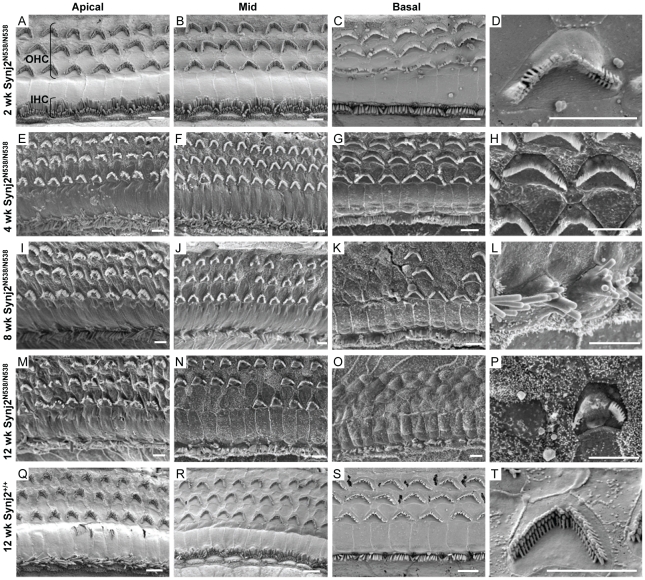
Loss of function of Synj2 leads to cochlear hair cell loss. Ultrastructural analysis using scanning electron microscopy of cochlear hair cells in *Synj2* ^N538K/N538K^ and *Synj2* ^+/+^ mice. Apical, mid and basal cochlear turns were examined at 2, 4, 8 and 12 weeks of age. As early as 2 wks of age, signs of hair cell degeneration are seen in outer hair cells of the basal cochlear turn (Panel D). At 4 weeks, the majority of basal outer hair cells appear sunken with fused stereocilia (Panel H). Mid cochlear inner hair cells show stereocilia fusion at 8 weeks (Panel L). Fused stereocilia or complete loss of hair bundles is common in mid region outer hair cells at 12 weeks (Panel P). Panel T shows the structure of a normal outer hair cell. OHC, outer hair cells; IHC, inner hair cells. Scale bar: 4 µm.

The process of degeneration of the organ of Corti around the time of the onset of hearing impairment was examined in more detail by ultrastructural analysis using scanning electron microscopy (SEM). Cochleae from 2, 4, 8 and 12 week old mice were analyzed (Fig. 5). Degeneration of outer hair cells and fusion of stereocilia was apparent as early as two weeks in the basal cochlear region (Fig. 5C and 5D). At four weeks of age (Fig. 5E, 5F, 5G, 5H) signs of degeneration were seen in all turns of the cochlea, although most evident at the basal turn (Fig. 5G, 5H). Both inner and outer hair cells showed fusion of the stereocilia, and basal outer hair cells appear sunken and withdrawn from neighboring supporting cells (Fig. 5H). At eight weeks of age, hair cell degeneration was more pronounced. In the basal cochlear turn, the majority of outer hair cell hair bundles were missing, and those that remained showed extensive stereocilia fusion (Fig. 5K). Some hair bundles were also missing from the outer hair cells of the mid cochlear turn (Fig. 5J). At 12 weeks of age, the basal region of the cochlea was completely void of outer hair cell hair bundles and inner hair cells showed extensive stereocilia fusion and loss (Fig. 5O). Degeneration of apical and mid regions of the cochlea also progressed; the majority of hair cells exhibited fused stereocilia, and many outer hair cells lacked hair bundles (Fig. 5M, 5N). At each time-point investigated, degeneration of the outer hair cells appeared more advanced and severe than that of the inner hair cells of the same cochlear region.

### No Loss of Nerve Fibers in *Synj2* ^N538K/N538K^ Mice

**Figure 6:**
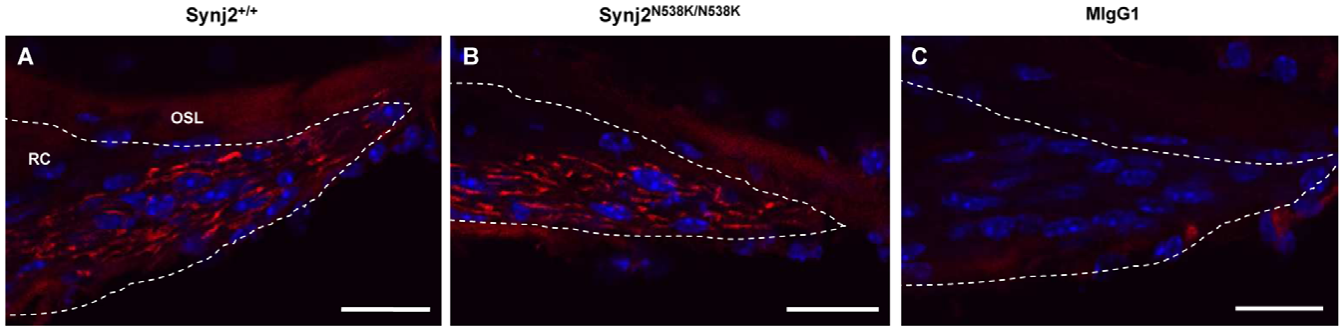
Neurofilament expression in Rosenthal's canal. Expression of NF-L in cochlear nerve fibres (red) of *Synj2* ^+/+^ (Panel A) and *Synj2* ^N538K/N538K^ mice (Panel B) within the Rosenthal's canal (dotted line). Isotype control showing no non-specific expression (Panel C). OSL, osseas spiral lamina; RC, Rosenthal's canal. Scale bar: 20 µm.

Hair cells are often the primary site of damage in sensorineural hearing loss, with the degeneration of cochlear nerve fibers a secondary consequence [24]. The loss of these fibers has been reported in a number of mouse models for hearing loss [25], [26], and in mice exposed to harmful levels of noise [27]. To determine whether loss of cochlear nerve fibers underlies the hearing loss in *Synj2* ^N538K/N538K^ mice, antibodies were used to detect neurofilament NF-L and neurofilament 200kD (NF-H) in cryosections of 12 week *Synj2* ^+/+^ and *Synj2* ^N538K/N538K^ mice. NF-L expression was evident in fibers of the cochlear nerve in wild-type and *Synj2* ^N538K/N538K^ mice, when compared to an isotype control (Fig. 6). No apparent differences in the levels of expression of NF-L or NF-H (data not shown), or in the number of fibers, were observed between *Synj2* ^+/+^ and *Synj2* ^N538K/N538K^ mice by this method. Estimating neuronal fiber density or individually counting peripheral axons would however provide a more sensitive and accurate method to determine any discrete differences between genotypes.

### *Synj2* is Expressed in the Hair Cells, but not the Spiral Ganglion of the Inner Ear

**Figure 7:**
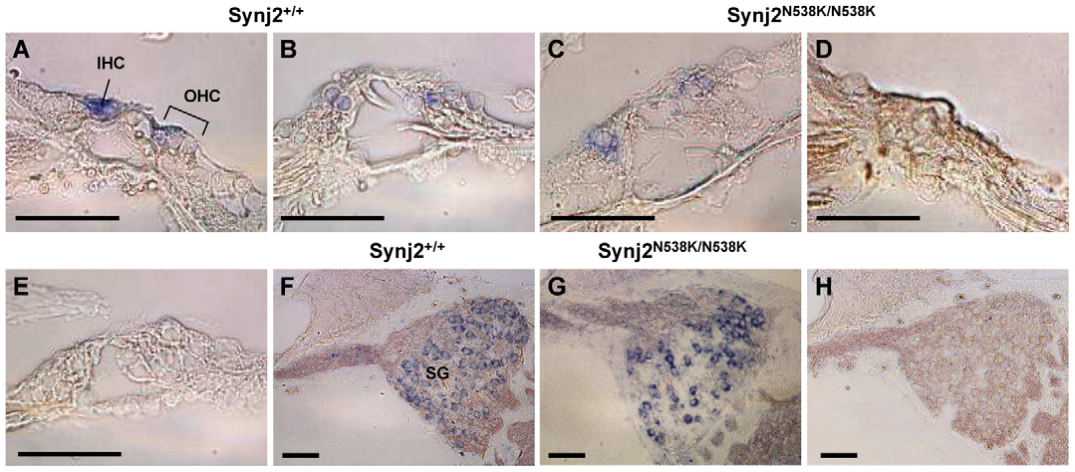
*Synj2* but not *Synj1* is expressed in the hair cells of the mouse cochlea. *Synj2* expression (blue staining) within the inner and outer hair cells of *Synj2* ^+/+^ mice at 4 weeks (Panel A) and 12 weeks (Panel B), and *Synj2* ^N538K/N538K^ mice at 4 weeks (Panel C) and 12 weeks (Panel D). Control *Synj2* sense probe staining is shown in Panel E. *Synj1* expression in *Synj2* ^+/+^ (Panel F) and *Synj2* ^N538K/N538K^ (Panel G) spiral ganglion in 12 week old mice. Control sense *Synj1* probe staining is shown in Panel H. IHC, inner hair cells; OHC, outer hair cells; SG, spiral ganglion. Scale bar: 50 µm.

As Synj2 mutant mice exhibit deafness, *in situ* hybridization was performed to determine the site of *Synj2* expression within the cochlea (Fig. 7). In wild-type *Synj2* ^+/+^ adult mice, *Synj2* mRNA expression was detected in the inner and outer hair cells (Fig. 7A and 7B), but was not observed in the spiral ganglion. In *Synj2* ^N538K/N538K^ mutants, *Synj2* expression was detected at 4 weeks of age, but was not observed at 8 and 12 weeks following the degeneration of hair cells and collapse of the organ of Corti (Fig. 7C and 7D). Expression of the highly related paralog enzyme, *Synj1*, was investigated in 12 week old mice. In contrast to *Synj2*, expression of *Synj1* was not detected in hair cells, but was highly expressed in spiral ganglion cells of adult wild-type mice (Fig. 7F). *Synj1* expression was still evident in remaining spiral ganglion cells in the *Synj2* ^N538K/N538K^ mouse, despite obvious degeneration of the spiral ganglion neurons (Fig. 7G). No differences in *Synj1* and *Synj2* expression levels or patterns were observed when heterozygotes and wild-type mice were compared. We conclude that *Synj2*, but not *Synj1,* is expressed in the hair cells of the inner ear.

## Discussion

This study has identified a novel regulator of hair cell survival, which is essential for normal hearing, as the phosphoinositide signal regulating enzyme, Synj2. We have identified a mouse mutant with a non-syndromic, recessively inherited, progressive hearing loss due to an ENU-induced mutation in the *Synj2* gene. *Synj2* ^N538K/N538K^ mice exhibit a rapidly progressive hearing loss and at 12 weeks of age are severely deaf. The ABR hearing thresholds in heterozygous *Synj2* ^N538K/+^ mice are similar to those in wild-type littermates and the hearing loss in *Mozart* is therefore recessively inherited. *Synj2* ^N538K/N538K^ mice do not exhibit circling or head tossing behavior, and respond normally in the simple trunk curl test. We therefore conclude that they do not have vestibular dysfunction. Scanning electron microscopy of cochleae from postnatal day 4 (P4) *Synj2* ^N538K/N538K^ mice suggests that hair cells have developed normally (data not shown). However, they show signs of degeneration as early as two weeks of age. Initial stages of degeneration include fusion of stereocilia followed by complete loss of hair bundles and ultimately loss of hair cells, which is most profound at the basal cochlear region. Outer hair cells begin to degenerate earlier than inner hair cells and show the most profound degeneration. As the neurofilament numbers are normal in *Synj2* ^N538K/N538K^ mice, it is likely that loss of such fibers is not the underlying cause of hearing loss in these mice. It therefore appears that Synj2 is essential for hair cell survival and normal hearing.

Synj2 is a member of the inositol polyphosphate 5-phosphatase family, of which ten mammalian enzymes have been characterized, many of which exhibit diverse roles in physiological function. Synj1 and Synj2 are two closely related 5-phosphatases [10], [28], that contain an N-terminal catalytic domain (Sac1) involved in hydrolysis of additional phosphoinositides [29] followed by a central 5-phosphatase domain that hydrolyses the 5-position phosphate from the inositol ring of PtdIns(4,5)P_2_, PtdIns(3,4,5)P_3_ and Ins(1,4,5)P_3_ (Fig. 1). Mice with mutations in *Synj1* die shortly after birth due to neurological complications [14] associated with the accumulation of clathrin-coated vesicles in the nerve terminals as a consequence of failure to degrade PtdIns(4,5)P_2_. Deafness or inner ear abnormalities have not been reported in *Synj1* mice and *Synj1* is not expressed at detectable levels in adult mouse hair cells. However, expression of *Synj1* has been previously reported in adult zebrafish hair-cell transciptome using amplified RNA [30], and more recently in developing hair cells of zebrafish larval by *in situ* hybridization analysis [31], and synaptosomes from 2 week old chicken cochleae using microarray and western blot analysis [32]. Synj1 has been implicated in hair cell function in zebrafish. Synj1-deficient *nrc* and *comet* zebrafish mutants revealed impaired vestibular–ocular defects with normal startle acoustic response [31], [33], [34]. Examination of the *Synj1* ^Q296X^ mutant zebrafish neuromast hair cells showed blebbing near synaptic ribbons. It was shown that this is a consequence of an imbalance between exo- and endocytosis and dependent on Cav1.3 calcium channel activity. Electrophysiological investigations revealed decreased number of readily releasable vesicles and far fewer reserve pool vesicles, as well as defective phase locking of afferent activity. Synj1 was proposed to play a critical role in facilitating vesicle recycling by affecting the number of vesicles released and the timing of release [31]. The absence of detectable *Synj1* expression in hair cell in 12 week old mice in this study could reflect the limited sensitivity of the technique utilized and/or indicate a restricted temporal expression of *Synj1* in mouse hair cells. Synj2 is a much less characterized enzyme, and our *Synj2* ^N538K/N538K^ mice are the only reported mouse strain with a mutation in the *Synj2* gene (the gene affected in a mouse mentioned in [35] appears to be in *Synj1* and not in *Synj2*). Therefore our study represents the first association of *Synj2* with a disease phenotype.

The mutation in Synj2, p.N538K, leads to significant loss of catalytic activity towards two of its substrates, PtdIns(3,4,5)P_3_ and PtdIns(4,5)P_2_. The Synj2 Asn^538^ residue is highly conserved in species as divergent as Drosophila and yeast. Whisstock *et al* mutated the corresponding asparagine to an alanine in the mouse 43 kDa inositol polyphosphate 5-phosphatase (encoded by the *Inpp5a* gene) and showed that this residue is essential for catalytic hydrolysis of Ins(1,4,5)P_3_ [22].

Phosphoinositides are components of eukaryotic cell membranes that act as signaling molecules, regulating many cellular processes including actin polymerization, apoptosis and vesicular membrane trafficking [14], [36], [37], [38], [39], [40], [41], [42]. Mutations in genes affecting phosphoinositide metabolism can therefore be expected to have important consequences for cell function and survival. *Mozart*'s relatively mild phenotype is somewhat unexpected, especially since the p.Asn538Lys *Synj2* mutation appears to significantly ablate 5-phosphatase activity in enzyme assays. Although we are unable to yet explain the mild phenotype of the *Synj2* ^N538K/N538K^ mice, we can suggest several possible explanations: first, the mutated Synj2^N538K^ enzyme may have enough residual phosphatase expression to allow normal cell function, except in the hair cells where our *in situ* results suggest a higher level of Synj2 activity is present and may be required for cell function; second, Synj2 has several important domains that function independently from the 5-phosphatase activity. These functions are retained in our mutant mouse and the lack of Synj2 5-phosphatase activity could be largely compensated for in mice (apart from within the cochlea) by other 5-phosphatases [43]; third, Synj2^N538K^ protein may bind its substrate PtdIns(4,5)P_2_ and/or PtdIns(3,4,5)P_3_, but not release it, thereby affecting an inner ear specific function, or fourth, low levels of Synj1, undetectable by *in situ* hybridisation, may compensate for the lack of Synj2 5-phosphatase activity in the mutant in a temporal fashion. In support of this hypothesis the detection of phosphoinositide biosensors, AKT/PH-GST or PLCδ∼1PH-GST, by immunohistochemistry in tissue sections from wild-type versus *Synj2* ^N538K/N538K^ mice did not identify any detectable difference in their distribution or levels.

It is known that inositol phosphates and phosphoinositides do play an important role in hearing. PtdIns(4,5)P_2_ is hydrolyzed by phospholipase C to generate Ins(1,4,5)P_3_ which mobilizes intracellular calcium. Ins(1,4,5)P_3_ and ATP release in cochlear cells leads to the propagation of regenerative Ca^2+^ waves, intracellular free Ca^2+^ that spreads through cells supporting the sensory cells [6], [44], [45], [46]. This intracellular signaling affects important cellular processes [47], some of which might be essential for correct sound processing [46]. A number of connexin 26 mutations have been reported in people with hearing loss, causing impaired Ins(1,4,5)P_3_ connexin hemichannel permeability [7], [9], therefore demonstrating that this is a process essential for propagation of Ca^2+^ waves and normal cochlear function [8]. Mechanisms for sensing damage to sensory hair cells from acoustic trauma have also been linked to the availability of functional Ins(1,4,5)P_3_ stores in supporting cells [48]. Hirono *et al* showed that PtdIns(4,5)P_2_ itself plays an essential role in hair cell transduction and adaptation in frogs [49]. Mice with mutations in the transmembrane phosphatidylinositol phosphatase gene *Ptprq* [50] develop abnormal hair cell stereocilia, most likely from altered actin organization, or turnover, as well as changes to membrane trafficking due to changes in phosphoinositide levels in hair cells [51]. Phosphoinositides are also involved in mediating ototoxicity of aminoglycoside antibiotics [52], [53] through a process involving formation of reactive oxygen species [54]. PtdIns(3,4,5)P_3_ has been associated with a decline of survival capacity in ageing outer hair cells [55]. PtdIns(4,5)P_2_ has been shown to play a significant role in regulating ion channel activity, including potassium channels such as KCNQ [56]. Notably mutations in the potassium channel gene, KCNQ4, which is expressed in the hair cells of the inner ear, underlie DFNA2, a subtype of autosomal dominant progressive, high-frequency hearing loss. Mice with loss of function of KCNQ4 exhibit normal vestibular function, but have progressive hearing loss associated with loss of the outer hair cells, reminiscent of *Synj2* ^N538K/N538K^ *Mozart* mice [57]. Therefore it is tempting to speculate that loss of Synj2 function in degrading PtdIns(4,5)P_2_ leads to altered KCNQ channel activity and hair cell degeneration.

Mutations in other lipid phosphatases that regulate phosphoinositide signaling molecules have been associated with abnormal neurological function. Recently Inpp4a, an enzyme that degrades PtdIns(3,4)P_2_, the lipid product of 5-phosphatase hydrolysis of PtdIns(3,4,5)P_3_, was identified as a suppressor of neuroexcitatory cell death [58]. *Pale tremor* mice have light fur pigmentation, severe tremors and abnormal gait due to a mutation in the *Fig4* gene, coding for a PtdIns(3,5)P_2_ 5-phosphatase [59]. In humans, mutations in *FIG4* cause Charcot-Marie-Tooth type 4J neuropathy [59]. We therefore investigated if *Synj2* ^N538K/N538K^ mice had more subtle neurological features by measuring nerve conductance in the sciatic nerve, as well as conducting a primary behavioral screen. However, homozygous mutant mice did not show any statistically significant differences from *Synj2* ^N538K/+^ littermates.

Mouse models of disease are often excellent models of human conditions. Analysis of human DNA samples to determine if mutations in the *SYNJ2* gene cause hearing loss in humans is in progress.

Although studies are beginning to provide an insight into the role of some phosphoinositides in hearing loss, the specific mechanisms of how the p.Asn538Lys mutation in *Synj2* ^N538K/N538K^ mice causes a progressive, non-syndromic hearing loss, and the role of phosphoinositides in this process is not yet understood. Further characterization of our novel *Synj2* ^N538K/N538K^ mouse model will provide new insights into the functional and structural features of the auditory system, and the role of *Synj2* and phosphoinositides in the auditory process.

## Materials and Methods

### The *Mozart* Mouse

The *Mozart* mouse was generated at the Australian Phenomics Facility (APF) as part of a mouse ENU mutagenesis program (http://www.australianphenomics.org.au/services.html). Male C57BL/6 mice were treated weekly for 3 weeks with 100 mg/kg N-ethyl-N-nitrosourea (ENU). To identify recessive phenotypes, G2 siblings were mated to generate consanguineous G3 offspring homozygous for ENU mutations. Recessive inheritance was validated in the *Mozart* strain by backcrossing deaf mice onto wild-type C57BL/6 mice followed by brother-sister matings, to confirm that approximately 25% of the offspring had a hearing loss. All mouse procedures were approved by the Royal Children's Hospital Animal Ethics Committee, RCH AEEC #A488 and #A585.

### Hearing Testing of Mice

G3 offspring of ENU-mutagenised mice were initially screened for hearing loss using a clickbox that elicits a Preyer reflex or startle response in hearing mice. The clickbox produces an 18.9 kHz burst of 106 dB SPL at a distance of 10 cm (Institute of Hearing Research, Nottingham, UK). It provided a convenient, fast, low-cost and thus suitable high-throughput phenotypic screen. Mice that failed the initial clickbox hearing test had a more detailed assessment of possible hearing loss using an evoked auditory brainstem response (ABR) test (AEP, Bio-logic Systems Corp.). Subdermal active, reference and ground electrodes were placed at the vertex, ventrolateral to the left ear and the abdomen, respectively, of the anaesthetized mouse. Specific auditory stimulus in the form of broadband clicks was delivered in a range of decibel sound pressure levels (dB SPL) and the ABR recorded. Subsequent offspring were then screened by ABR to obtain a hearing profile for each genotype. Mice were also screened for the presence of vestibular dysfunction, identified by the display of hyperactivity that manifests either as circling, head tossing/tilting and/or star gazing behaviour.

### Mapping and Mutation Detection

Deaf *Mozart* mice were outcrossed with the CBA/H mapping strain and the *Mozart*/CBA/H offspring were then crossed to each other to produce affected F2 generation mice for homozygosity mapping. Genomic DNA (isolated by phenol/chloroform extraction from tail biopsies) from 20 affected mice were analyzed by genome wide scans using 120 microsatellite markers (AGRF, Australia). Fine mapping was performed using Amplifluor-based SNP assays (APF, Australia) on 69 additional affected mice. For statistical analysis we used the normal approximation to the binomial test for proportions of homozygous C57BL/6 genotypes (hearing loss mutants), to map the deafness loci. In regions of linkage, the proportion of homozygous C57BL/6 genotypes would approach 1.0 whilst at unlinked loci this proportion would be 0.25. Linkage intervals were examined for known or putative deafness genes using the UCSC genome browser. PCR primers were designed to amplify and sequence the coding regions, intron-exon boundaries and the 5′ and 3′ untranslated regions of candidate genes. Primer sequences are available upon request. Sequencing was performed using BigDye terminators V3.1 (ABI).

### Site-directed Mutagenesis and 5-Phosphatase Enzyme Assay

*Synj2* or *Synj2* ^N538K^ 5-phosphatase domains were sub-cloned into the pGEX-KG vector in-frame with the GST tag. Expression of recombinant GST-tagged proteins was induced in *E.coli* by incubation of 50 ml cultures of cells with 0.1 mM IPTG for 3 hours at 30°C. Cell pellets were suspended in 5 ml GST extraction buffer (50 mM HEPES (pH 7.5), 150 mM NaCl, 1 mM EDTA, 10 mM MgCl_2_) + 1% Triton X-100, 1 mM PMSF, 0.2 µg/ml aprotinin, 0.2 µg/ml leupeptin, 1 mM benzamidine and incubated at 4°C for 90 minutes with gentle agitation. Lysates were centrifuged at 13,000 g for 10 minutes then the supernatants incubated with 100 µl pre-washed glutathione Sepharose^TM^ 4B beads overnight at 4°C with rotation. Samples were washed 6 times in ice-cold GST extraction buffer then PtdIns(3,4,5)P_3_ or PtdIns(4,5)P_2_ 5-phosphatase assays were performed on the affinity precipitates. For PtdIns(3,4,5)P_3_ 5-phosphatase assays, 50 µl PtdIns([^32^P]3,4,5)P_3_ prepared as described [60] was added to the affinity precipitates together with 4 µl 20 x kinase buffer (400 mM HEPES (pH 7.5), 100 mM MgCl_2_, 20 mM EGTA). Reactions were incubated at 37°C for 20 minutes and the lipids extracted and analyzed by TLC as previously described [60]. For PtdIns(4,5)P_2_ 5-phosphatase assays, 5 µg PtdIns(4,5)P_2_ (Echelon Biosciences, Salt Lake City, UT) was added to 40 µl of the affinity precipitates together with 55 µl 1 x kinase buffer. Reactions were incubated at 37°C for 30 minutes then 20 µl supernatants and 80 µl malachite green (Echelon Biosciences) were added to a 96 well plate in duplicate, incubated at room temperature for 15 minutess and the absorbance measured at 655 nm. Phosphate release was quantitated according to the manufacturer's instructions.

### Cochleae Isolation

Adult mice were anaesthetized with isoflurane and culled by cervical dislocation according to the National Health and Medical Research Council Australian code of practice for the care and use of animals for scientific purposes. Intact cochleae were surgically removed using a posterolateral approach, as described previously [61]. Brains and other tissues were dissected from mice when needed for further analysis.

### Hematoxylin and Eosin (H&E) Staining

Cochleae were isolated from 4, 8 and 12 week *Synj2* ^+/+^, *Synj2* ^N538K/+^ and *Synj2* ^N538K/N538K^ mice. Tissues were fixed in 4% paraformaldehyde (PFA) for 1–1.5 hours at room temperature with rotation, washed 3 x 5 minutes in TBS, and then transferred to 10% EDTA for 4–7 days at 4°C to decalcify. Decalcified cochleae were washed through a series of increasing sucrose solutions and incubated overnight at 4°C in 30% sucrose/PBS, then transferred to OCT (Sakura, USA) and left overnight at 4°C to allow the OCT to fully penetrate the tissue. Cochleae were embedded in OCT, sectioned (10 µm) using a Leica cryostat and placed onto Superfrost Plus microscope slides (Thermo Scientific, USA). Sections were dried at room temperature and stored at −20°C until required. Sections were then subjected to standard H&E staining.

### Immunohistochemistry

Cochleae were prepared as described above for H&E staining. Cryosections were thawed at room temperature for a minimum of 30 minutes, washed 3 x 10 minutes in PBS and then incubated with 0.3% Triton X-100 for 20 minutes to permeabilise the tissue. Slides were then briefly washed in PBS and incubated with Image-iT FX Signal Enhancer (Invitrogen) for 30 minutes in a humidified chamber at room temperature to reduce background staining. Slides were washed a further 3 x 10 minutes with PBS, and pre-blocked in Blocking Solution (30% goat serum (GS), 2% BSA and 0.3% Triton X-100 in PBS) for 30 minutes before incubation with primary antibody in 1% GS, 0.1% Triton X-100 in PBS overnight at 4°C. Slides were washed for 3 x 10 minutes in PBS and incubated for a further 2 hours in the dark with an Alexa Fluor 594-conjugated goat anti rabbit IgG antibody or a 488-conjugated donkey anti mouse IgG antibody (both 1∶1500; Molecular Probes) in 1% GS/PBS. Slides were washed an additional 3 x 10 minutes and mounted with Prolong® Gold antifade reagent with DAPI (Invitrogen). Slides incubated with primary antibodies omitted the incubation step with anti-GST antibody. Fluorescence was analysed using a Leica TCS SP2 laser scanning confocal microscope, and images generated with Leica Confocal Software (Leica Microsystems).

Primary antibodies used were anti-neurofilament NF-L monoclonal antibody (1∶500; Covance), anti-neurofilament 200kD polyclonal antibody (1∶500; Chemicon), purified mouse IgG1 (1∶500; Invitrogen) and rabbit serum (1∶500; Sigma).

### *In Situ* Hybridization

Cochleae were isolated and then processed for *in situ* hybridization as previously described [61]. Digoxigenin-labelled (DIG RNA labelling mix, Roche) anti-sense and sense riboprobes for *Synj1* and *Synj2* were generated by nested PCR, using cDNA made from RNA isolated from snap frozen brains of wild-type mice. For *Synj2* the following primers were used to create a 542 bp riboprobe: Synj2 ExtF 5′-GCTTTTGAAAGGCACATGGT-3, Synj2 ExtR 5′-AGCATCCGTCCTTTGTCTGT-3′, Synj2 IntF T7 5′-GAGTAATACGACTCACTATAGGAAGTTGCTCTGGGCTTCTTG-3′, Synj2 IntR Sp6 5′-TGTATTTAGGTGACACTATAGAACAGTAGCTTGATGGCCTCCT-3′. For *Synj1*, the following primers were used to create a 619 bp riboprobe: Synj1 ExtF: 5′-GCAGTTTGCACAC TTTGGTG-3′, Synj1 extR: 5′-GGGCTTTTGGGTGCTTCTAT, Synj1 IntF T7: 5′-GTGTAATACGACTCACTATAGGGAAGCTCTGCCTTGAA-3′, Synj1 IntR Sp6: 5′-TATATTTAGGTGACACTATAGACTAGCAGGCGCAGGACTC-3′. Riboprobe hybridization was visualized using an Anti-Digoxigenin-Alkaline Phosphatase conjugated antibody (Roche) and BCIP/NBT (Roche) color reaction.

### Scanning Electron Microscopy (SEM)

For SEM analysis, inner ears were isolated, the round and oval windows were cleared and the cochlear apex was pierced to allow thorough perfusion of the fixative. Inner ears were fixed in 2.5% glutaraldehyde in 0.1 M sodium cacodylate buffer (pH 7.4) with 3 mM CaCl_2_ for 3 hours at room temperature. Samples were then washed in PBS and further dissected by removing the bony shell, stria vascularis, Reissner's and tectorial membranes, to reveal the cochlear sensory epithelium. Four, 8 and 12 week cochlear specimens were processed with the OTOTO method (osmium tetroxide/thiocarbohydrazide) adapted from Hunter-Duvar *et al* [62], then dehydrated in ethanol, critical point dried (CPD 20, Bal-Tec), mounted on stubs using conductive silver paint and viewed using a Hitachi FE S-4800 Scanning Electron microscope. Cochleae from 2 week old mice, and some additional 12 week mice, were coated with osmium tetroxide for 2.5 hours, washed, dehydrated in ethanol, critical point dried (CPD 030, Bal-Tec), mounted on stubs using silver paint, gold sputter coated (Edwards S150B sputter coater, ∼10 nm) and viewed using a Philips XL30 FE Scanning electron microscope.

### Nerve Conductance Tests

Peripheral nerve conduction was assessed in two ways by electromyography (EMG). The sciatic nerve was stimulated and the time it took for the compound muscle action potential to be generated was measured. These same recordings were also investigated for a second peak, indicating that sciatic stimulation activated Ia sensory fibers in the nerve and evoked a muscle action potential via their monosynaptic connections with sciatic motor neurons in the spinal cord. The second method of examining nerve conduction was to determine nerve conduction velocity directly by taking the difference in the latency of the first peak when changing the point of sciatic nerve stimulation by a known distance. By subtracting the latencies we cancel out any delays in the generation of the EMG response caused by neuromuscular transmission and generation of the muscle action potential, and are left with the difference in latency caused by the change in distance. This is an accurate way to determine if there are differences in conduction velocity in different groups of mice, although the velocities measured by this “whole nerve” stimulation approach differ from some single fibre measurements.

### Behavioral Tests

Primary behavioral screens of 12 wild-type, 12 heterozygous and 12 homozygous mutant *Mozart* mice, consisting of locomotor, light-dark, Y-maze, tail suspension, hot plate, rotarod and marble burying tests, were done at the Integrative Neuroscience Facility at the Florey Institute, Melbourne (http://www.hfi.unimelb.edu.au/inf/).
